# Monetary reward speeds up voluntary saccades

**DOI:** 10.3389/fnint.2014.00048

**Published:** 2014-06-20

**Authors:** Lewis L. Chen, Y. Mark Chen, Wu Zhou, William D. Mustain

**Affiliations:** ^1^Department of Otolaryngology and Communicative Sciences, University of Mississippi Medical CenterJackson, MS, USA; ^2^Department of Ophthalmology, University of Mississippi Medical CenterJackson, MS, USA; ^3^Department of Neurobiology and Anatomical Sciences, University of Mississippi Medical CenterJackson, MS, USA; ^4^Department of Neurology, University of Mississippi Medical CenterJackson, MS, USA

**Keywords:** reward, saccadic velocity, voluntary saccade, nasal-temporal asymmetry, sensorimotor priming

## Abstract

Past studies have shown that reward contingency is critical for sensorimotor learning, and reward expectation speeds up saccades in animals. Whether monetary reward speeds up saccades in human remains unknown. Here we addressed this issue by employing a conditional saccade task, in which human subjects performed a series of non-reflexive, visually-guided horizontal saccades. The subjects were (or were not) financially compensated for making a saccade in response to a centrally-displayed visual congruent (or incongruent) stimulus. Reward modulation of saccadic velocities was quantified independently of the amplitude-velocity coupling. We found that reward expectation significantly sped up voluntary saccades up to 30°/s, and the reward modulation was consistent across tests. These findings suggest that monetary reward speeds up saccades in human in a fashion analogous to how juice reward sped up saccades in monkeys. We further noticed that the idiosyncratic nasal-temporal velocity asymmetry was highly consistent regardless of test order, and its magnitude was not correlated with the magnitude of reward modulation. This suggests that reward modulation and the intrinsic velocity asymmetry may be governed by separate mechanisms that regulate saccade generation.

## Introduction

Reward contingency is essential for behavior modification and learning. Many studies have documented that reward signals are processed through the network associated with dopamine neurons (Schultz et al., 1997; Hikosaka et al., 2000; Schultz, 2006; Kobayashi and Schultz, 2008; Bromberg-Martin and Hikosaka, 2009; Basso and Sommer, 2011; Glimcher, 2011). It has been shown that the reward-modulated signals in the caudate nucleus facilitate saccade generation by dis-inhibiting the pre-saccadic burst activity in the superior colliculus, a structure that is critical for the generation of saccades (Hikosaka and Wurtz, 1985a,b; Sparks, 2002; May, 2006). This suggests that reward expectation is a useful variable to probe the cognitive control of saccades.

Two recent non-human primate studies have provided independent psychophysical evidence showing that reward expectation sped up saccades (Takikawa et al., 2002; Chen et al., 2013). In these studies, the animals were operant conditioned to perform a series of unrewarded and rewarded saccades either in the same block of trials (Takikawa et al., 2002) or within the same trial (Chen et al., 2013). The findings of these studies unequivocally indicate that saccadic velocity was a movement variable directly modulated by reward expectation, and not a by product of changes in saccadic amplitude. This implication is consistent with a dopamine depletion study, in which an interruption of the reward-related circuitry in the basal ganglia significantly disrupted the main sequence, i.e., amplitude-velocity relationship, such that saccadic velocity was reduced even if the amplitude was identical (Kato et al., 1995). That is, the decline of dopamine signal resulted in a decline of saccadic peak velocity. These lines of evidence strongly indicate that the control of saccadic peak velocity was tightly linked to the dopamine-associated reward circuitry via the basal ganglia. Such control resulted in speeding up or slowing down saccades via the circuitries of saccadic generation (Sparks, 2002; Hikosaka et al., 2006).

The organization of non-human primates' brain is similar to that of human's. One may be curious as to whether comparable reward modulation can be demonstrated in human. For human studies, audio/visual feedback and monetary reward often serve as positive reinforcers. There is evidence that the blood-oxygen-level dependent signals in the brain regions were correlated with the amount or delay of the received reward (Kable and Glimcher, 2007, 2010; McClure et al., 2007; Gregorios-Pippas et al., 2009; Schultz, 2013; van den Bos and McClure, 2013; van der Vegt et al., 2013; Rodriguez et al., 2014). These observations are consistent with those observed in non-human primate studies, which showed that the activities of dopamine neurons varied systematically with the amount or delay of the received reward (Kobayashi and Schultz, 2008; Bromberg-Martin and Hikosaka, 2009; Hwang et al., 2009). There is also evidence that socially relevant visual stimuli, such as face images, produced reward-like neuronal responses (Hayden et al., 2007), speeding up orienting saccades (Xu-Wilson et al., 2009). Monetary reward presumably activates the reward circuitries in human, similar to how the juice reward works in monkeys (Takikawa et al., 2002; Hikosaka et al., 2006; Schultz, 2006; Chen et al., 2013).

This study was set out to investigate the above question. We developed a methodology to address the issue of amplitude-velocity coupling without sacrificing the amplitude sensitivity. Our results showed that monetary reward indeed sped up human saccades. In addition, we examined the reward modulation of temporal and nasal saccades (Robinson, 1964; Collewijn et al., 1988). We found that the magnitude of reward modulation was not correlated with the magnitude of the velocity asymmetry.

## Materials and methods

### Subjects

Seven healthy subjects (4 female and 3 male, aged 18–52 years old) participated in this study. All subjects had normal or corrected-to-normal vision with no known neurological and psychiatric disorders. None of the authors was among the subjects reported in this study. All subjects received verbal/written instructions and were provided with a written consent in compliance with the Institutional Review Board of the University of Mississippi Medical Center.

### Recording of gaze positions

Horizontal eye positions were recorded from subjects' right eye using a Skalar IRIS infrared limbus tracker (Delft, The Netherlands; spatial resolution: 0.1°) (Reulen et al., 1988) at 500 Hz. Subjects were seated 52-cm in front of a computer monitor (27”, resolution: 1080 × 800 pixels, 96 DPI, refresh rate: 75 Hz). Subjects' head position and orientation were restricted by the combination of a chin rest and a bite-bar. The height of the chin rest was adjusted, such that the subjects' eyes were leveled with the center of the monitor. The right eye of the binocularly viewing subject's was centered with the screen. Visual stimulus display, behavioral scheduling, and data recording were controlled by a real-time data acquisition system (Beethoven; Ryklin, Inc.), which guaranteed a temporal resolution of 1 ms (for details, see Chen et al., 2013).

### Behavioral procedures

**Figure 1:**
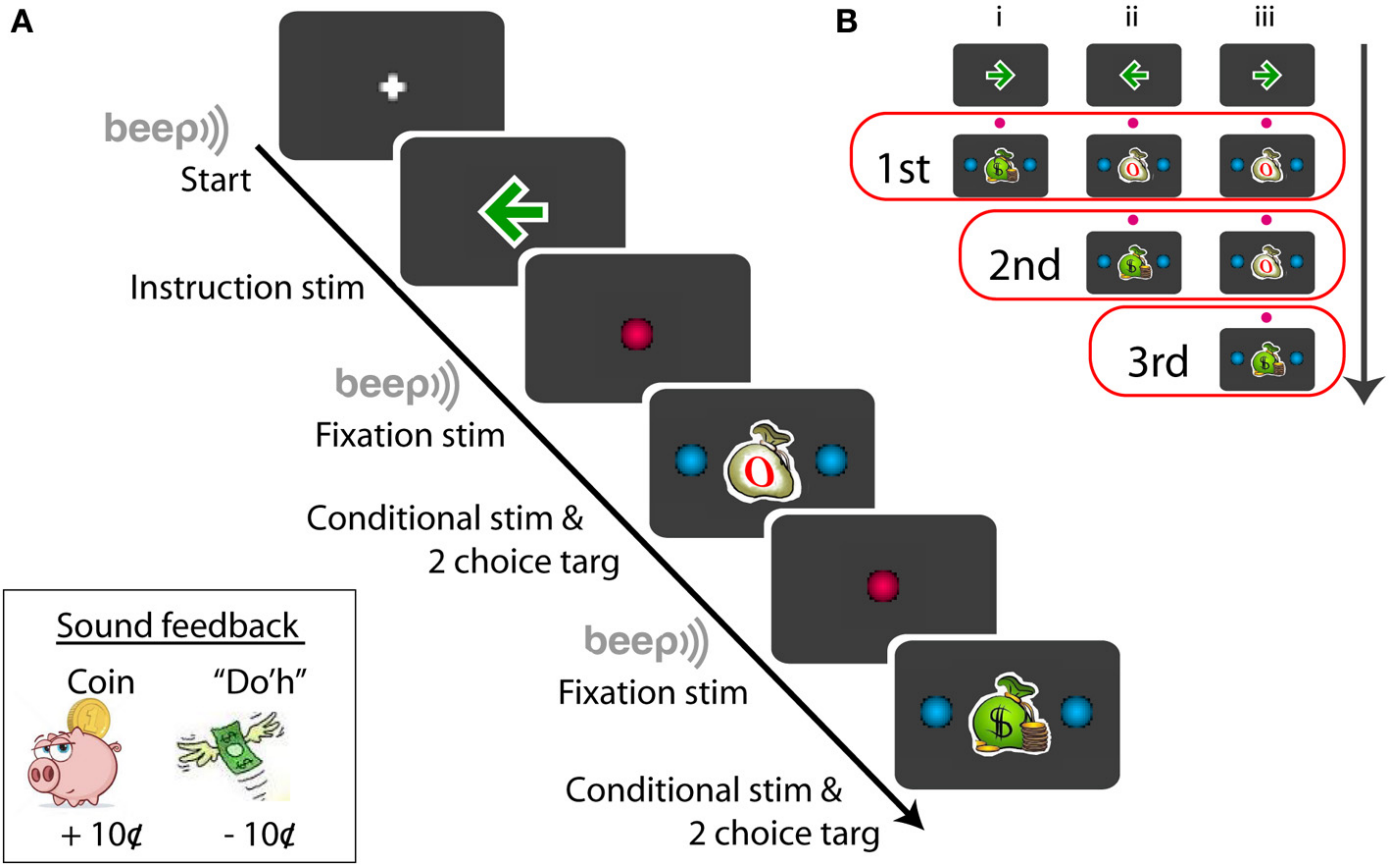
**Schematic example of the sequence of the conditional saccade task (A) and the conditional stimuli displayed for the series of tests (B)**. Symbols not drawn in scale; for details, see Materials and Methods. The red open boxes in **(B)** include the stimuli presented for the given (first, second, or third) test order.

Prior to each recording, subjects were told about the conditional stimulus-response procedure. Subjects were told that they would be paid for making a correct saccade in response to a congruent conditional stimulus (money bag, Figure 1) and they would *not* be paid for making a correct saccade in response to an incongruent stimulus (empty bag, Figure 1). Each subject was given 5–10 trials to practice before the recording began.

Figure 1A illustrates a procedural schematic of the conditional saccade task. Each trial started with a white plus sign (1.2°) displayed on the center of a gray screen (RGB: 60/60/60). As soon as the subject fixated at the plus sign, a green arrow (RGB: 0/155/0, dimension: 1.7°) was displaced at the fixation location for 600 ms. The arrow served as the instruction signaling the target direction (left vs. right) that was associated with monetary reward. The arrow was then replaced by a red fixation dot (RGB: 255/0/80, 1.2° in diameter) for 600–700 ms. Then, a “beep” tone signaled that a saccade test was to follow.

The test stimuli consisted of a conditional stimulus (a green money bag or a white empty bag), displayed at the fixation position, and 2 choice targets, placed symmetrically and horizontally from the conditional stimulus. The subjects' job was to review the conditional stimulus and to make a saccade to one of the choice targets within 1500 ms. The conditional stimuli were either a congruent stimulus (a green money bag, dimension: 1.7°) or an incongruent stimulus (a white empty bag, dimension: 1.7°) (Figure 1A). The choice targets were 2 blue dots (RGB: 0/175/240, 1.2° in diameter), placed 6–11° eccentric from the conditional stimulus (For subject F1, the targets were displayed at ±6, ±7, ±8, ±9, ±10, and ±11°; for other subjects, ±6, ±7, ±8, ±9, and ±10°). The correct choice target for the congruent stimulus was in the same direction as the instruction arrow, whereas that for the incongruent stimulus was opposite from the instruction arrow. Note that the test stimulus stayed illuminated until the subject made a saccade or the maximum response time (1500 ms) expired. Subjects were told to make just one saccade. There was no time pressure for the subjects to respond quickly. Target fixation was imposed for the initial 80-ms out of the entire duration (200 ms) of target display. This was implemented so that eye blinking following fixation would not abort the trial. Follow the same reasoning, the (red) central fixation was imposed for the initial 200-ms out of the entire duration (600–700 ms). The fixation “window” was ±4° from the designated coordinate.

Each trial consisted of up to 3 series of saccade tests (Figure 1B). Each saccade test repeated the steps of “fixate -> review instruction -> fixate -> review conditional stimulus -> make a choice.” There were 3 trial types, randomly interleaved in the same block (Figure 1B). The first trial type (Figure 1B,i) consisted of a single saccade test: a money bag; the second trial type (Figure 1B,ii) consisted of 2 series of saccade tests: an empty bag followed by a money bag; and the third trial type (Figure 1B,iii) consisted of 3 series of saccade tests: an empty bag, followed by an empty bag, and followed by a money bag. The arrow directions, trial types, and target eccentricities were randomly selected for each trial in order to minimize subjects' anticipation and adaptation. Given a flawless task performance, the overall reward rate was pre-determined: 33, 50, and 100% for the first, second, and third saccade test, respectively. However, based on the *post-hoc* subject interview, none of the subjects was aware of the difference in the reward probability between the first and second saccade tests.

Sound feedback was provided during the task (Figure 1A, lower-left inset). After the subject made a correct saccade in response to the congruent stimulus, the sound of coin drop (at the cash register) was played. This signaled that a coin (10¢) was deposited to the subject's bank. No sound was played after a correct saccade was made based on the incongruent stimulus, and no coin was deposited. To discourage making errors, the subject were penalized for making an *incorrect* saccade in response to either a congruent or an incongruent stimulus. In this case, Homer Simpson's “Do'h” voice was played and a coin was removed from the subject's bank (−10¢). The errors varied from subject to subject, typically 1–5% of the data. Aborted trials, including failure to fixate (e.g., excessive blinking) or failure to respond before the maximum response time expired, were not penalized.

Each trial lasted 2.5–4.0 s, and the inter-trial interval was set at 1.2 s. The subjects were given approximately 30-s of break after completing each block of 100 successful rewarded saccades. They were told to close their eyes and relax without removing themselves from the chin rest. A recording session typically lasted 30 min, up to an hour maximum.

### Data analyses

Off-line analyses were performed using an in-house program on a Windows platform. Eye positions were smoothed using a 5-point parabola filter (Chen and Walton, 2005; Chen et al., 2013). Saccade onset and offset were defined when movement velocity exceeded or fell below a threshold of 30°/s. Movements were displayed on screen for visual inspection before measurement. Eye movements with double peaks in velocity profiles (<1% of data), likely resulting from eyelash artifacts or blinking, were removed from further analysis.

Only successful trials in which all saccades within the trials were correctly performed were included in the present study. Usually, the subject's performance improved rapidly in a few trials. The present analyses included only the correct trials after the subject's performance reached a considerably stable level, i.e., 5 consecutive successful trials. The first 3 trials immediately following each break were excluded from the analysis.

Saccadic velocity has been shown to be tightly coupled with amplitudes (Bahill et al., 1975; Chen et al., 2013). Hence, we quantified saccadic velocities based on assorted amplitude bins. The saccadic velocity of a given amplitude bin was initially assigned as the averaged velocity value of the bin. To guard against the estimate irregularity resulting from data binning, the velocity estimate was then averaged with those of two adjacent (forward and backward) bins. This 3-point moving average was applied only once and only to the velocity-amplitude series under the same experimental treatment, prior to further analyses.

The percent change of peak velocity (%C_PV_) across amplitude bins was quantified as:

$$\%{\text{C}}_{\text{PV}}=\left(\sum_{i}^{n}\frac{{\text{PV}}_{A_{i}}-{\text{PV}}_{B_{i}}}{{\text{PV}}_{B_{i}}}\right)\text{​}/n$$

where PV_*A_i_*_ is the average peak velocity (PV) at bin *i* for saccade A, while PV_*B_i_*_ is the average PV at bin *i* for saccade B. For example, for the computation of reward modulation, saccade A is rewarded (R) saccade (i.e., PV_*A_i_*_ = PV_*R_i_*_), whereas saccade B is unrewarded (UR) saccade (i.e., PV_*B_i_*_ = PV_*UR_i_*_). Each bin width is 0.5°. *n* is the total number of amplitude bins, each of which consists of valid measures obtained from saccades A and B.

The change of saccadic PV (C_PV_) across amplitude bins was quantified as:

$${\text{C}}_{\text{PV}}=\frac{\sum_{i}^{n}\left({\text{PV}}_{A_{i}}-{\text{PV}}_{B_{i}}\right)}{n}$$

where PV_*A_i_*_ is the average PV at bin *i* for saccade A, while PV_*B_i_*_ is the average PV at bin *i* for saccade B. For example, for the computation of the nasal-temporal velocity asymmetry, saccade A is the saccade of the temporal (T) direction (i.e., PV_*A_i_*_ = PV_*T_i_*_), whereas saccade B is the saccade of the nasal (N) direction (i.e., PV_*B_i_*_ = PV_*N_i_*_). Each bin width is 0.5°. *n* is the total number of amplitude bins, each bin of which consists of valid measures of saccades A and B.

Statistical analysis was performed using Statistica (StatSoft Co.; Snedecor and Cochran, 1989). Data were described as mean ± s.e.m. unless otherwise specified.

## Results

**Figure 2:**
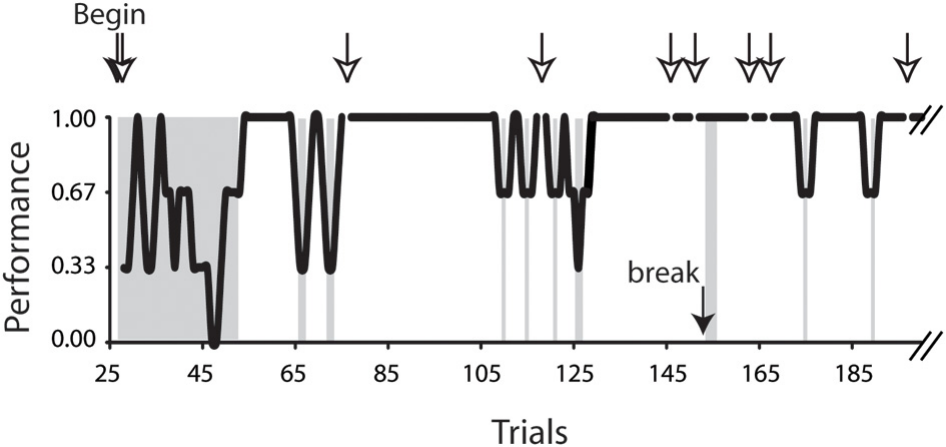
**Task performance of the conditional saccade task**. Data were obtained from subject F3, plotted up to trial 190 out of a total of 411 trials. The performance was computed as 3-point moving averages across trials. Open arrows indicate the aborted trials; the filled arrow indicates one of the breaks during the recording session. The trials in the gray patches included the beginning trials (trials 28–53), 3 trials immediately after a break (trials 154–156), and all error trials (for details, see Materials and Methods). These trials were excluded from the analyses reported in this study.

The analyses were conducted on 6.0–10.5° horizontal saccades obtained from 7 (4 female and 3 male) subjects. Only the saccades of successful trials were included in the present analysis. Figure 2 plots a typical task performance of the conditional saccades (Chen and Wise, 1995a). A trial was considered successful if all of the series of saccadic choices were correct. A successful trial was counted as 1, whereas an unsuccessful one, 0. The performance is illustrated as 3-point moving averages of the success scores. It was typical that the subject's performance improved rapidly after a brief period of practices. As the performance stabilized, i.e., reaching the level of 5 consecutive successes, the saccades of successful trials were selected for further analyses. The success rate was 91, 80, 71, 80, 77, 85, and 84% for subject F1, F2, F3, F4, M5, M6, and M7, respectively.

### Reward modulation on the saccadic amplitude-velocity relationship

**Figure 3:**
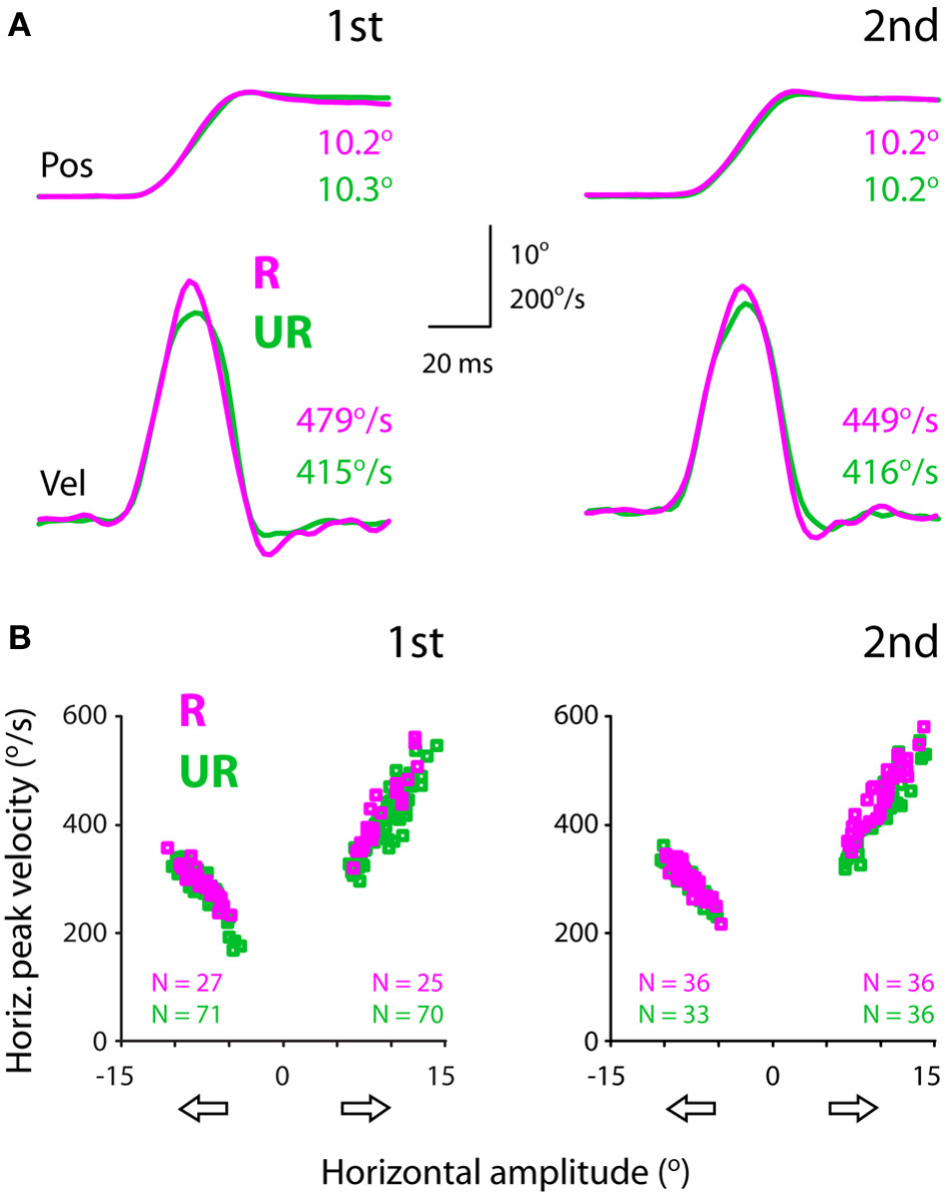
**Exemplar position (top) and velocity (bottom) traces (A) and the amplitude-velocity main sequence relationship between rewarded and unrewarded saccades, showing that the saccadic velocities were relatively higher in rewarded than unrewarded saccades, independent of the amplitude-velocity coupling (B)**. The amplitudes (abscissa) of rightward and leftward saccades are assigned as positive and negative values, respectively. The data were obtained from subject F1, separated for saccadic directions (open arrows) and test order (first: left, second: right).

Figure 3A showed exemplar position (top) and velocity (bottom) traces of rightward saccades. It can be noted that these saccades had comparable amplitudes (10.2–10.3°). However, rewarded saccades (first test: 479°/s; second test: 449°/s) were faster than unrewarded saccades (first test: 415°/s; second test: 416°/s).

Figure 3B plots saccadic velocities as a function of amplitudes (abscissa) for all successful saccades from the same subject. Even though there existed an intimate amplitude-velocity coupling, rewarded rightward saccades were in general faster than unrewarded rightward saccades. This apparent reward modulation persisted in both tests (first: left plot; second: right plot), confirming the impression of individual velocity traces (Figure 3A). The question is how one quantifies the reward modulation embedded in the main sequence, i.e., amplitude-velocity relationship. This point will be dealt with in the next section.

Note that this subject's rightward (temporal) saccades were faster compared to leftward (nasal) saccades (Figure 3B). For instance, 10° rightward saccades had a peak velocity of ~420°/s, whereas 10° leftward saccades had a relatively lower peak velocity, ~320°/s. This idiosyncrasy of saccadic velocity preference has been reported previously (Robinson, 1964; Collewijn et al., 1988) and will be addressed in the latter section.

### Quantification of reward modulation of saccadic velocities

**Figure 4:**
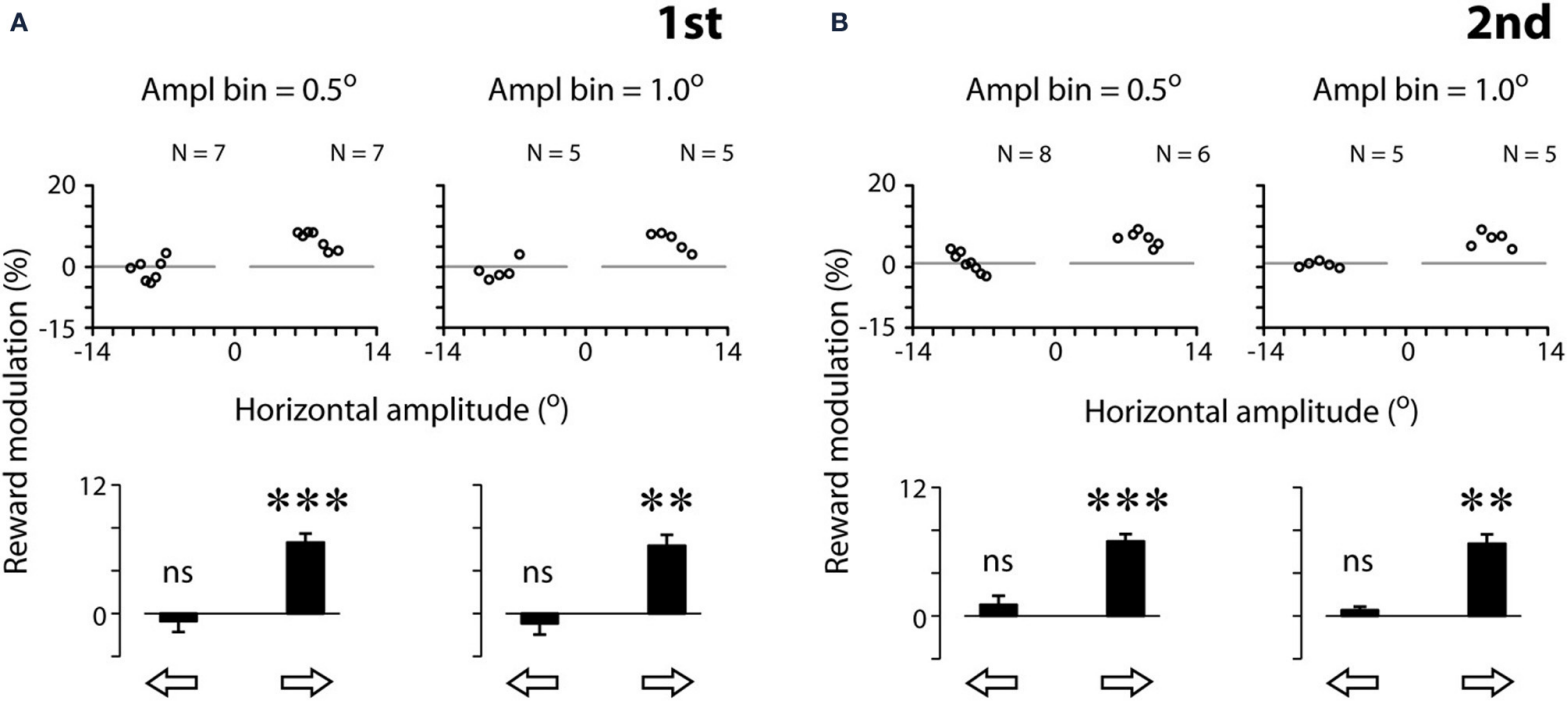
**Quantification examples of reward modulation of saccadic velocities based on 2 different amplitude bin widths [0.5° (left panels) and 1.0° (right panels)], separated by the first (A) and second (B) tests**. Reward modulation is plotted as the percent velocity change between rewarded and unrewarded saccades across the amplitude bins in the scatter plots (Equation 1, see Materials and Methods). The modulation across all scatter plot data per saccadic direction is plotted as a bar chart. Note that the data plotted were analyzed following the procedure of 3-point moving average (see Materials and Methods). The results *without* the procedure of 3-point moving average procedure are as follows. First test, 0.5°: 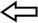: −0.4 ± 4.3 (*N* = 7, *P* > 0.05), 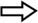: 7.9 ± 4.7 (*N* = 7, *P* < 0.01); First test, 1.0°: 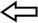: −0.1 ± 3.0° (*N* = 5, *P* > 0.05), 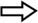: 7.0 ± 3.4 (*N* = 5, *P* < 0.05); second test, 0.5°: 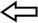: 1.2 ± 5.1° (*N* = 8, *P* < 0.05), 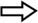: 7.9 ± 6.1 (*N* = 6, *P* < 0.05); second test, 1.0°: 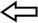: −1.4 ± 3.3° (*N* = 5, *P* > 0.05), 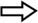: 7.0 ± 3.4° (*N* = 5, *P* < 0.05). Note that the resilience of the reward modulation remained in agreement with the data plotted, even though there was a significant increase in data variance without the 3-point moving average procedure. *P* < 0.05^*^; *P* < 0.01^**^; *P* < 0.001^***^, n.s.: *P* > 0.05. Data from subject F3.

Figure 4 quantifies the effect of reward expectation on saccadic velocities. The scatter plots show the individual data point for each amplitude bin width (left panels: 0.5°; right panels: 1.0°; see Materials and Methods). Note that the results based on bin width of 0.5° and 1.0° were in general agreement with one another. We tested other bin widths ranging from 0.1 to 1°, and the same pattern of results was found; hence we only present the data of bin width of 0.5 and 1.0°. This is not surprising as the eye tracker has a resolution of 0.1°, rendering noisy data analysis at 0.1° bin width (data not shown). For proper analysis, as a convention, the measurement resolution should be set at ≥3x of that of the equipment. Based on these reasons, we opted to apply 0.5° amplitude bin width for the rest of the analyses.

For this subject (F3), all data points of rightward saccades were above zero, reflecting that reward expectation significantly sped up the saccades across all amplitude bins (Figure 4, middle scatter plots; one-sample *t*-test, 2 tail, *P* < 0.001 for both tests). In contrast, the data points of leftward saccades were distributed around zero, reflecting the lack of modulation (*P* > 0.05 for both tests). The bar charts show the reward modulation separated for saccadic directions (open arrows; Figure 4).

As can be noted in Figure 4, a reduction of amplitude bin width did not increase proportionally the number of valid data points in the scatter plots. For example, a 10-fold reduction of amplitude bin width (from 1.0 to 0.1°) led to approximately 2-fold increase of data points. This is because that each bin must contain both rewarded and unrewarded data to be counted as a valid data point in the plot (see Materials and Methods). The number of valid data points was not increased in proportion with the decrease of bin width.

**Figure 5:**
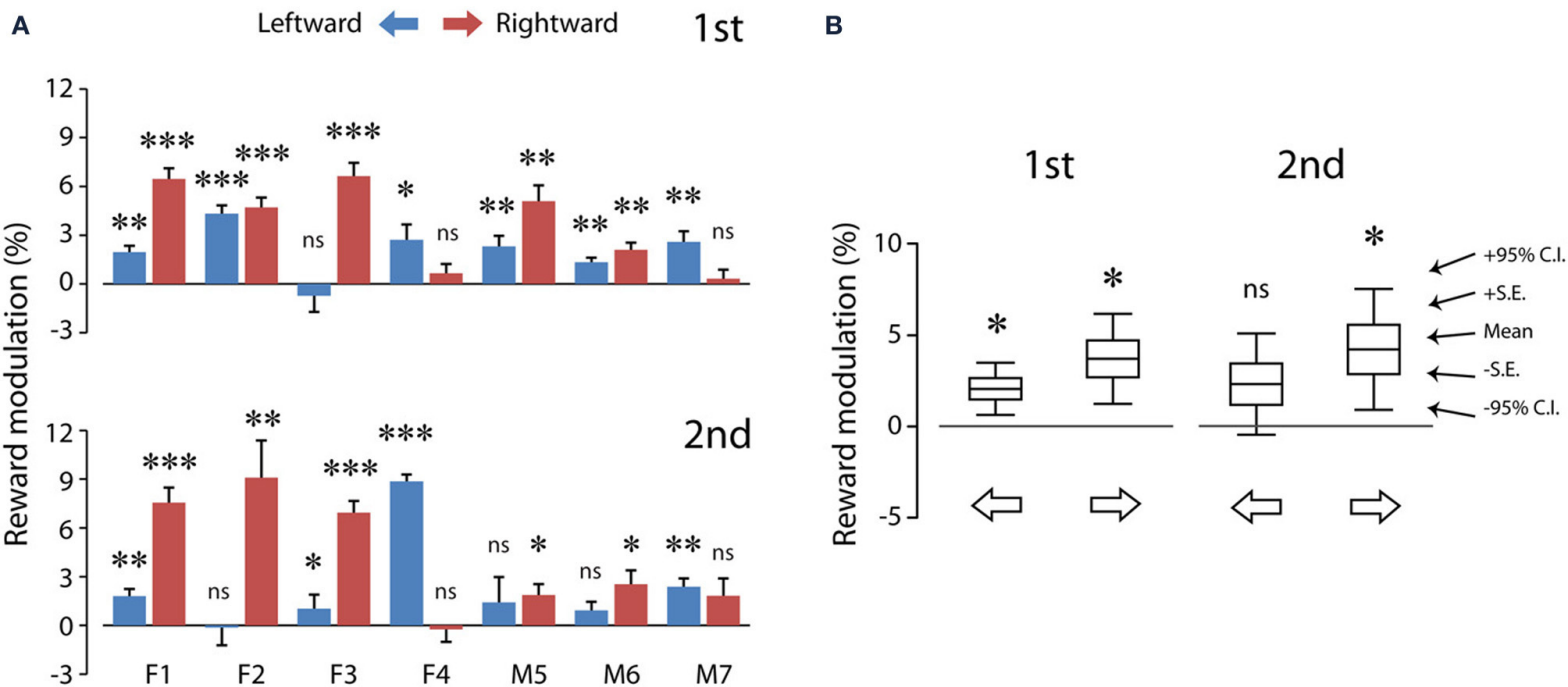
**The average reward modulation of saccadic velocities separated for saccadic directions of each subject (A) and the population averages of reward modulation across subjects (B)**. Data in **(A)** were quantified using the method shown in Figure 3 (see Materials and Methods; Equation 1), separated for test order (first: top; second: bottom). The data from each subject are plotted in paired color bars, for leftward (blue) and rightward (magenta) saccades, respectively. Note that, despite the individual differences in **(A)**, the reward modulation was significant in the population averages in **(B)**. *P* < 0.05^*^; *P* < 0.01^**^; *P* < 0.001^***^, n.s.: *P* > 0.05. C.I.: confidence interval.

Figure 5A shows the reward modulation of the saccadic velocities of individual subjects. Consider the first test (Figure 5A, top). Reward expectation significantly sped up leftward saccades (one-sample *t*-test, 2 tail, *P* < 0.05) in all subjects (up to 4.3%) except subject F3 (−0.7%). In addition, reward expectation significantly sped up rightward saccades (one-sample *t*-test, 2 tail, *P* < 0.05) in all subjects (up to 6.6%) except subjects F4 (0.7%) and M7 (0.3%). In other words, monetary reward sped up voluntary saccades at least in one of the horizontal directions. Note that reward modulation did not negatively impact the saccadic velocities in these subjects, consistent with the previous findings in the non-human primate studies (Takikawa et al., 2002; Chen et al., 2013).

Similar results were obtained during the second test (Figure 5A, bottom). Reward expectation significantly sped up leftward saccades (one-sample *t*-test, 2 tail, *P* < 0.05) in all subjects (up to 8.9%) except subjects F2 (−0.1%), M5 (−1.4%) and M6 (0.9%). In addition, reward expectation significantly sped up rightward saccades (one-sample *t*-test, 2 tail, *P* < 0.05) in all subjects (up to 9.1%) except subjects F4 (−0.2%) and M7 (1.8%). That is, monetary reward sped up saccades, even though these rewarded saccades were presumably pre-primed by a preceding unrewarded saccade.

Table 1 shows the analysis based on the peak velocity changes between rewarded and unrewarded saccades for each subject (see Materials and Methods; Equation 2). The results of Table 1, including the level of statistical significance, were consistent with the reward percentage results obtained from Figure 5.

**Table 1:** **Reward-associated velocity modulation computed as the average peak velocity (PV) changes (mean ± s.e.m.) between rewarded and unrewarded saccades (see Materials and Methods; Equation 2)**.

	**Leftward saccade**			**Rightward saccade**		
	**PV change (°s)**	***N***	***P***	**PV change (°s)**	***N***	***P***
**FIRST TEST**						
F1	6.0±1.2	8	<0.01	25.9±3.3	8	<0.001
F2	13.4±1.5	9	<0.001	16.0±2.4	9	<0.001
F3	−2.2±2.8	7	n.s.	19.9±2.1	7	<0.001
F4	9.2±3.3	8	<0.05	2.7±2.2	9	n.s.
M5	5.8±1.6	9	<0.01	13.5±2.7	8	<0.01
M6	4.0±0.8	9	<0.01	7.6±1.7	9	<0.01
M7	9.3±2.1	9	<0.01	1.4±1.8	8	n.s.
**SECOND TEST**						
F1	5.5±1.3	8	<0.01	30.4±3.8	6	<0.001
F2	−0.1±3.5	8	n.s.	33.3±8.7	7	<0.01
F3	3.7±2.7	8	<0.05	22.5±2.1	6	<0.001
F4	29.0±2.4	8	<0.001	−0.3±2.9	9	n.s.
M5	3.1±3.7	7	n.s.	5.0±1.7	8	<0.05
M6	2.5±1.5	9	n.s.	8.9±3.0	8	<0.05
M7	8.5±1.8	9	<0.01	6.8±3.6	8	n.s.

Positive values indicate that rewarded saccades were faster than unrewarded saccades, whereas negative values, unrewarded saccades were faster than rewarded saccade. n.s.: p > 0.05.

Figure 5B shows the population summary for these subjects. The reward modulation during the first test was statistically significant for both leftward (2.1 ± 0.6%; 6.5 ± 1.9°/s, *N* = 7) and rightward (3.7 ± 1.0%; 12.4 ± 3.4°/s, *N* = 7) saccades (one-sample *t*-test, *P* < 0.05 for both). This indicates that the reward modulation was robust and detectable at the population averages. Nevertheless, the reward modulation during the second test was statistically significant only for rightward saccades (4.2 ± 1.3%; 15.2 ± 5.0°/s, *N* = 7; *P* < 0.05) and not for leftward saccades (2.3 ± 1.1%; 7.5 ± 3.7°/s, *N* = 7; *P* > 0.05). This is likely to result from increased data variance during the second test. There was no significant difference in the reward modulation between leftward and rightward saccades (2 dependent-sample *t*-test, *P* > 0.05 for both first and second tests).

**Figure 6:**
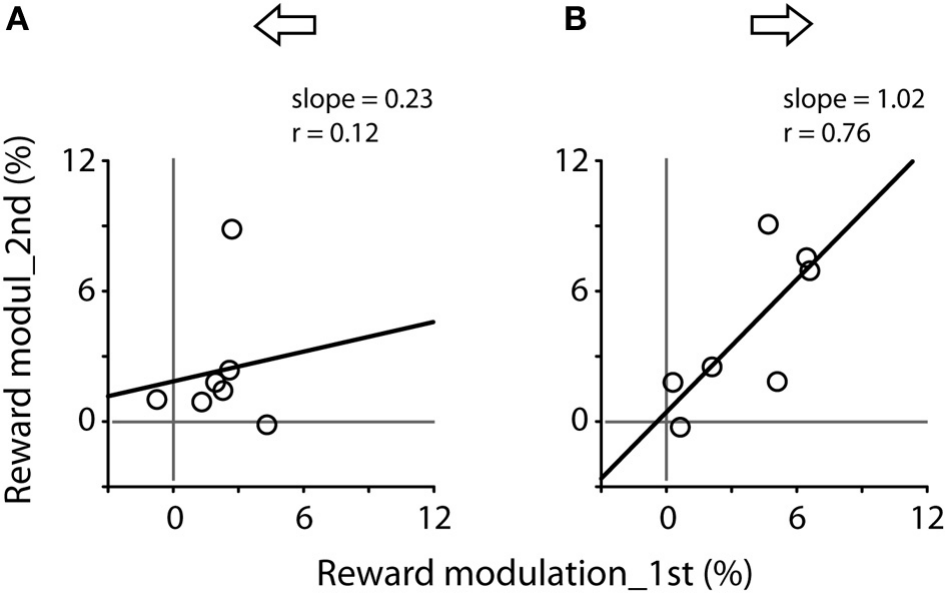
**Correlation of reward modulation between the first (abscissa) and second (ordinate) tests, separating for leftward (A) and rightward (B) saccades**. Data obtained from Figure 5A. Note the significant correlation for rightward saccades (Pearson correlation, *r* = 0.76, *P* < 0.05).

Figure 6 plots the correlation of reward modulation during the first test and that during the second test. There was a positive correlation for rightward saccades (B), whereas there was no apparent correlation for leftward saccades (A). Note that the right eyes were recorded from these subjects (see Materials and Methods).

### Nasal-temporal velocity asymmetry

**Figure 7:**
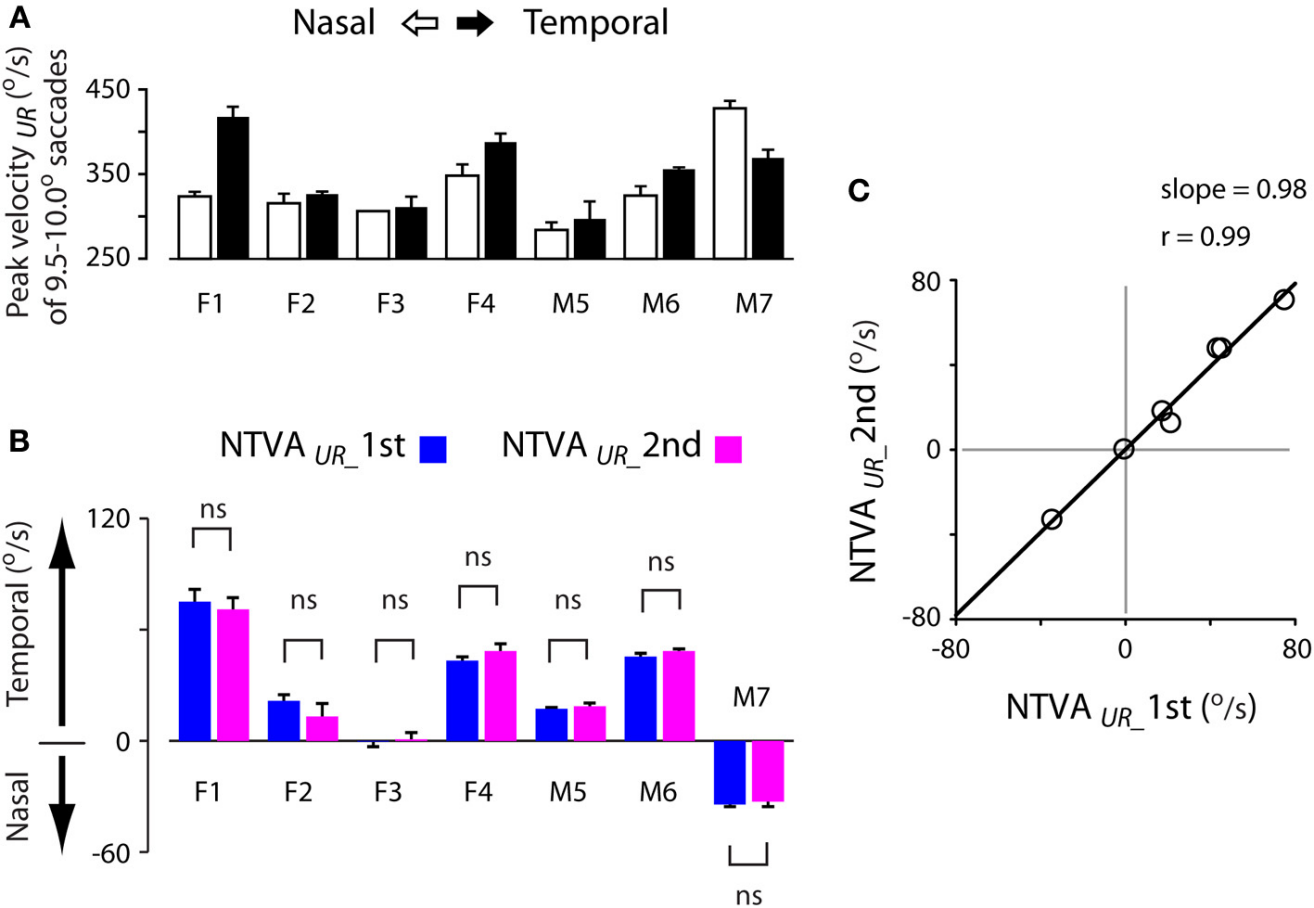
**Stability of the nasal-temporal velocity asymmetry (NTVA) of unrewarded saccades. (A)** Peak velocity data obtained from 9.5–10.0° unrewarded saccades during the first test of each subject, showing the saccadic velocity asymmetry across subjects (nasal saccades: open bars; temporal saccades: filled bars). **(B)** The NTVA of unrewarded saccades (NTVA_UR_) separated for the first (blue bars) and second (magenta bars) tests, showing the stability of the velocity asymmetry across these subjects (See Materials and Methods; Equation 2). Positive value indicates the velocity by which temporal saccades were faster than nasal saccades, and vice versa. **(C)** The correlation of NTVA between the first (abscissa) and second (ordinate) tests, showing a nearly 1-to-1 correlation between the repeated measures of unrewarded saccades. ns: *P* > 0.05.

Past studies have shown that saccadic velocities vary for abducting (temporal) or adducting (nasal) directions (Robinson, 1964; Collewijn et al., 1988). The main sequence relationship illustrated in Figure 3B agreed with this notion. Figure 7A shows an example of the velocity asymmetry across all subjects. The saccadic velocities were selected from 9.5–10.0° unrewarded saccades during the first test. Note that the velocity asymmetry varied significantly across these subjects.

The question is how stable was this velocity asymmetry? We plotted the nasal-temporal velocity asymmetry of unrewarded saccades (Figure 7B; see Materials and Methods; Equation 2). Positive values indicate that temporal saccades were faster than nasal saccades (subjects F1-M6), and vice versa (subject M7). There were 3 main points in this plot. First, subject F3 showed a lack of saccadic velocity asymmetry (one-sample *t*-test, 2 tail, *P* > 0.05 for either of the first or second test). This characteristic persisted across the two tests (2-sample *t*-test, 2 tail, *P* > 0.05). Second, all other subjects showed a significant nasal-temporal velocity asymmetry. Their saccadic velocities were on average 13–75°/s higher in the temporal or nasal direction than the opposite direction (*P* < 0.001 for either of the first and second test). Third, this intrinsic velocity asymmetry remained unchanged across the tests (2-sample *t*-test, 2 tail, *P* > 0.05 for all subjects). The cross-subject average change of the velocity asymmetry between the two tests was negligible (−0.0 ± 1.7°/s, 95% confidence interval: ±9.0°/s; *P* > 0.05).

The above picture was consistent with the normalized measures across amplitude bins (Table 2; see Materials and Methods, Equation 1). Again, the normalized measures showed that the saccadic velocity asymmetry persisted regardless of test order (2-sample *t*-test, 2 tail, *P* > 0.05 for all subjects). The cross-subject average of this measure was near zero (−0.1 ± 0.6, 95% confidence interval: ±3.1%; *P* > 0.05).

**Table 2:** **Nasal-temporal velocity asymmetry (NTVA) computed as the average percent velocity changes (mean ± s.e.m.) between temporal and nasal saccades (see Materials and Methods; Equation 1)**.

	**First test**			**Second test**		
	**NTVA (%)**	***N***	***P***	**NTVA (%)**	***N***	***P***
**UNREWARDED SACCADES**						
F1	19.7±1.1	9	<0.001	18.7±1.0	6	<0.001
F2	6.8±1.1	9	<0.001	3.8±2.0	8	n.s.
F3	−0.3±0.9	8	n.s.	0.0±1.2	7	n.s.
F4	12.0±0.6	9	<0.001	14.0±1.0	8	<0.001
M5	6.9±0.4	9	<0.001	7.2±0.8	6	<0.001
M6	13.2±0.4	9	<0.001	13.7±0.4	8	<0.001
M7	−10.5±0.5	9	<0.001	−10.2±0.7	8	<0.001
**REWARDED SACCADES**						
F1	22.0±1.3	7	<0.001	25.9±0.3	7	<0.001
F2	7.2±0.5	9	<0.001	12.7±1.5	8	<0.001
F3	6.4±1.0	6	<0.01	6.1±0.6	7	<0.001
F4	11.0±0.9	9	<0.001	7.9±0.5	8	<0.001
M5	9.4±0.8	8	<0.001	7.2±0.9	9	<0.001
M6	13.9±0.3	9	<0.001	14.4±0.5	9	<0.001
M7	−13.7±1.6	8	<0.001	−10.6±1.1	9	<0.001

Positive values indicate that temporal saccades were faster than nasal saccades, whereas negative values, nasal saccades were faster than temporal saccade. n.s.: p > 0.05.

Figure 7C shows the correlation of the nasal-temporal velocity asymmetry between the first (abscissa) and second (ordinate) tests. Note the nearly 1-to-1 correlation between the repeated measures of the velocity asymmetry of unrewarded saccades (Pearson correlation, *r* = 0.99, *P* < 0.001), suggesting that this velocity asymmetry was highly stable.

**Figure 8:**
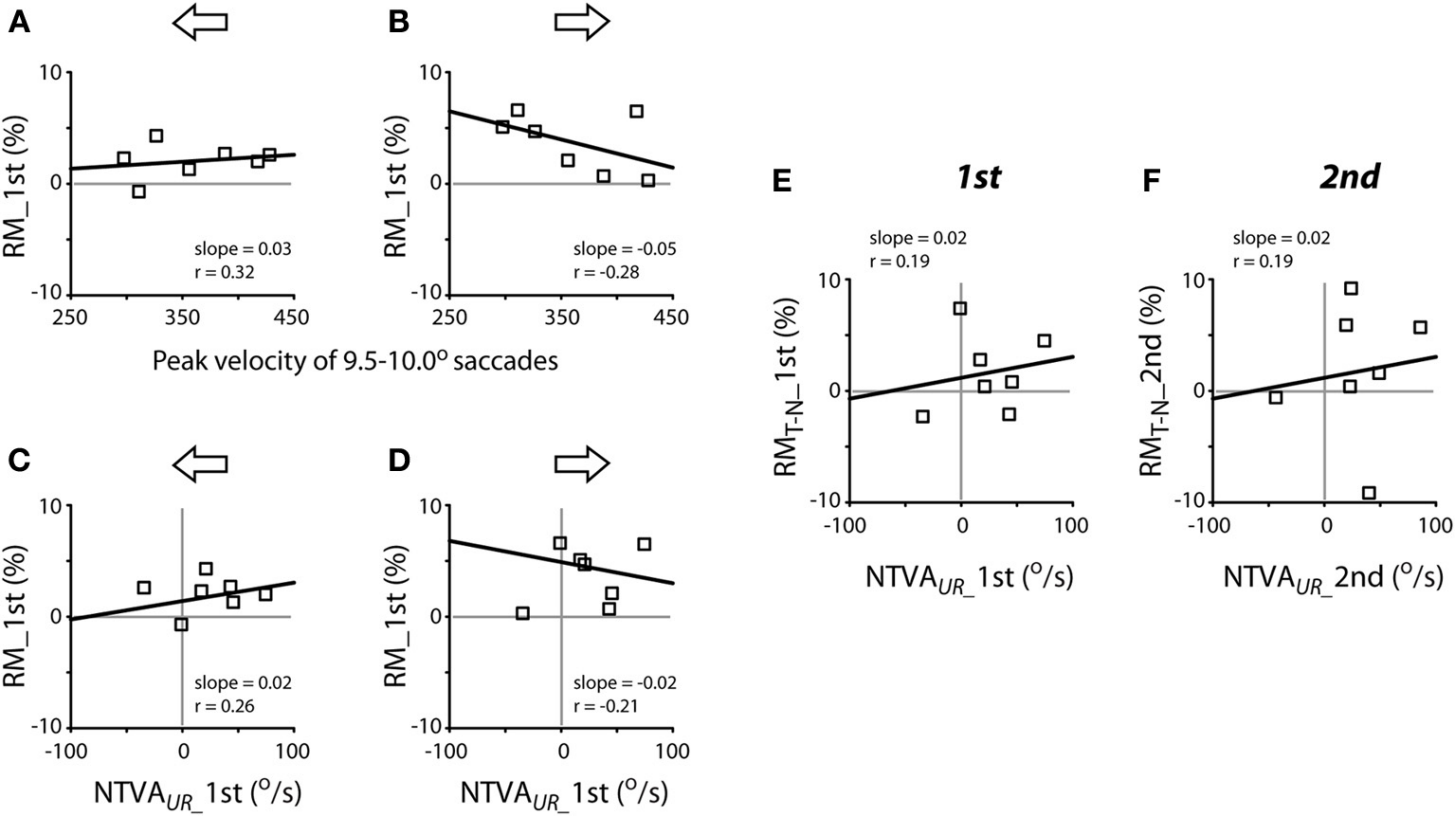
**Lack of apparent correlation between reward modulation (RM; ordinate) and individual subjects' peak velocities [abscissa; (A,B)] and the nasal-temporal velocity asymmetry [NTVA; abscissa, (C) and (D)] and lack of correlation between the differential reward modulation between temporal and nasal saccades and the nasal-temporal velocity asymmetry (E,F). (A,B)** Abscissa data obtained from Figure 7A; note that, in order to account for the representative velocity asymmetry from each subject, the velocities of temporal saccades were selected for subjects F1-M6, while the velocities of nasal saccades were selected for subject M7. **(C–F)** Abscissa data obtained from Figure 7B. The regression of **(C,D)** was done excluding the data of subject M7. Ordinate data obtained from Figure 5A.

The next question is whether the variability of peak velocities of unrewarded saccades or the magnitude of the intrinsic velocity asymmetry predicts the magnitude of reward modulation of saccadic velocity? Figure 8 plots the correlation between these variables, separated for leftward (A, C and E) and rightward (B, D and F) saccades. There was no apparent relationship between the peak velocities of a given amplitude of saccades (9.5–10.0° in this case) and the reward modulation during the first test (Pearson correlation, *P* > 0.05 for both leftward and rightward saccades; Figures 8A,B) or during the second test (slope = 0.02, ґ = 0.39, for leftward saccades; slope = −0.01, ґ = −0.21 for rightward saccades; *P* > 0.05 for both; data not shown). In other words, the subjects' tendency to make faster or slower saccades was not correlated with higher or lower reward modulation of saccadic velocity.

There was no apparent relationship between the magnitude of the nasal-temporal asymmetry and the reward modulation during the first test (Pearson correlation, *P* > 0.05; Figures 8C,D) or during the second test (slope = 0.03, ґ = 0.26, for leftward saccades; slope = −0.01, ґ = −0.07 for rightward saccades; *P* > 0.05 for both; data not shown). There was no apparent relationship between the magnitude of the nasal-temporal asymmetry and the differential reward modulation between temporal saccades and nasal saccades, either during the first or second test (Pearson correlation, *P* > 0.05; Figures 8E,F). That is, the magnitude of subjects' intrinsic velocity asymmetry was not correlated with the reward modulation of saccadic velocity.

## Discussion

The present study investigated the effect of monetary reward on the velocities of non-reflexive, visually-guided saccades. The subjects were financially compensated according to their saccadic response to a centrally-displayed rewarding, congruent stimulus, and they were not compensated for making the same response to an unrewarding, incongruent stimulus (Figure 1). There were three major findings. First, a methodology was developed to quantify the reward modulation of saccadic velocities independent of the amplitude-velocity coupling (Figures 3, 4). Based on this methodology, we found that monetary reward significantly sped up voluntary saccades up to 30°/s (Figures 3–5; Table 1). This suggests that monetary reward sped up saccades in human in a fashion analogous to how juice reward sped up saccades in monkeys. Second, for the rightward saccades of the right eye, the magnitude of reward modulation of the first test was positively correlated with that of the second test (Figure 6B), suggesting that, in spite of sensorimotor priming presumably resulting from consecutive saccades, the reward modulation was relatively consistent. Third, nasal-temporal velocity asymmetry was observed (Robinson, 1964; Collewijn et al., 1988). This intrinsic saccadic habit persisted regardless of test order (Figure 7), and the magnitude of the velocity asymmetry was not correlated with that of reward modulation (Figure 8). It's possible that the reward modulation mechanism is regulated independently from the intrinsic regulation of saccadic velocities.

### Reward speeds up saccades

Takikawa et al. (2002) and Chen et al. (2013) are the two non-human primate studies that provided independent psychophysical evidence showing that reward expectation sped up saccades. The two studies trained animals to perform a series of unrewarded and rewarded saccades either in the same block of trials (Takikawa et al., 2002) or within the same trial (Chen et al., 2013). The findings from the two studies and other neurophysiological studies unequivocally indicate that the expectation of reward indeed speeds up voluntary saccades. This is likely to result from the activation of the reward-related circuitry in the basal ganglia, which in turn influences the saccadic generation (Sparks, 2002; Hikosaka et al., 2006; Glimcher, 2011; Chen et al., 2013).

Our findings (Figures 3–5) showed that monetary reward increased the peak velocities of voluntary saccades in humans in a way analogous to how juice rewards sped up saccades in monkeys (Takikawa et al., 2002; Chen et al., 2013). To the best of our knowledge, the present study is the first investigation that addresses the question whether monetary reward modulates saccadic velocities in human. Most of our current knowledge regarding the neural processing of reward has been obtained from non-human primate studies. Hence, a brief review on the non-human primate literatures below is necessary, in order to highlight the background and significance of this line of studies. First of all, there is ample evidence indicating that dopamine neurons in the basal ganglia process the reward value of stimuli (Schultz et al., 1997; Kawagoe et al., 1998; Hikosaka et al., 2000; Tobler et al., 2005; Kobayashi and Schultz, 2008; Bromberg-Martin and Hikosaka, 2009; Matsumoto and Hikosaka, 2009; Glimcher, 2011; Levy and Glimcher, 2012). For example, when the reward contingency of a given target is abruptly altered, the dopamine neurons of the substantia nigra, as well as the directly linked caudate oculomotor neurons, immediately changed their neuronal correlates for the target (Kawagoe et al., 1998, 2004; Matsumoto and Hikosaka, 2009). This suggests that these neurons were involved in processing the reward value of a given target-action association, similar to the learning-related visual-oculomotor association neurons of the frontal lobes, which were intimately inter-connected within the cortico-basal ganglia loops (Chen and Wise, 1995a,b, 1996, 1997; Wise et al., 1996; Amador et al., 2000, 2004; Pasupathy and Miller, 2005; Ding and Hikosaka, 2006; Chen and Tehovnik, 2007). Several studies have shown a positive correlation between the neuronal activities of dopamine neurons in the basal ganglion and the peak velocities of saccades (Kato et al., 1995; Itoh et al., 2003). Itoh et al. (2003) carried out a study to account for the variability of saccadic velocity; the authors found that the discharge of dopamine-modulated caudate neurons was positively correlated with saccadic peak velocity. Kato et al. (1995) had demonstrated a causal link between the activity of dopamine neurons in the basal ganglia and the facilitation of saccadic velocity. The authors infused a neurotoxin, 1-Methyl-4-phenyl-1,2,3,6-tetrahydropyridine (MPTP), into the substantia nigra to selectively deplete saccade-related dopamine neurons. Two weeks after MPTP infusion, the animals showed a significant reduction of saccade frequency, amplitude, and velocity toward the side contralateral to the infusion site. In addition, the saccadic velocities were significantly reduced even if saccadic amplitudes remained unchanged. This indicates that the decrease in saccadic velocity was a direct consequence of dopamine depletion, not a byproduct of a decrease in saccadic amplitude.

The previous neurophysiological studies have indicated that dopamine-modulated oculomotor neurons in the basal ganglia regulate saccades through the superior colliculus (Wurtz and Goldberg, 1972; Schiller et al., 1980; Hikosaka and Wurtz, 1983, 1985a,b; Lee et al., 1988; van Opstal and van Gisbergen, 1990; van Opstal et al., 1995; Kawagoe et al., 1998; Sato and Hikosaka, 2002; Soetedjo et al., 2002; Hanes et al., 2005; Matsumoto and Hikosaka, 2009; Yasuda et al., 2012). For example, the superior colliculus– which received direct inhibitory inputs from the basal ganglia– exhibited a pre-saccadic burst of activity with a peak discharge rate positively correlated with saccadic peak velocity (van Opstal and van Gisbergen, 1990; van Opstal et al., 1995; Soetedjo et al., 2002). When the inhibitory synapses from the basal ganglia to the superior colliculus were blocked, saccadic velocities increased. In contrast, when the same synapses were promoted, saccadic velocities decreased (Hikosaka and Wurtz, 1985a,b; Lee et al., 1988). The above studies suggest that dopamine neurons in the basal ganglia modulate saccadic velocity in part through the oculomotor neurons in the superior colliculus.

Monetary reward has been used as a positive reinforcer in human studies (Kable and Glimcher, 2007, 2010; Schultz, 2013; van den Bos and McClure, 2013; Rodriguez et al., 2014). There is evidence that the blood-oxygen-level dependent signals of the areas targeted by dopamine innervation were correlated with the amount or the delay of the received reward (Kable and Glimcher, 2007, 2010; McClure et al., 2007; Gregorios-Pippas et al., 2009; Schultz, 2013; van den Bos and McClure, 2013; van der Vegt et al., 2013; Rodriguez et al., 2014). This observation was confirmed by non-human primate studies, in which the activities of dopamine neurons showed comparable decline function with the amount or the delay of the received reward (Kobayashi and Schultz, 2008; Bromberg-Martin and Hikosaka, 2009; Hwang et al., 2009). Hence, the monetary incentive indeed drives up the reward signal in brain. This explanation accounts for the general findings of this study. Note that socially relevant visual stimuli, such as face images, often serve as a positive reinforcer, producing reward-like neuronal responses (Kampe et al., 2001; Bray and O'Doherty, 2007; Hayden et al., 2007). These stimuli sped up orienting saccades, suggesting that socially rewarding targets may be associated with an intrinsic reward value that facilitates saccades (Xu-Wilson et al., 2009).

### Quantification of saccadic velocity independent of amplitude-velocity coupling

It is known since 1975 that saccadic peak velocity varies as a function of saccadic amplitude (Bahill et al., 1975; Baloh et al., 1975; Collewijn et al., 1988; Chen et al., 2013). As a result, variables associated with saccadic velocity cannot be easily quantified without proper control of saccadic amplitude. The methodology we developed in this study (Figure 4; see Materials and Methods) provided a few advantages regarding the quantification of saccadic velocities. One of the advantages is that the modulation of saccadic velocity was dissociated from saccadic amplitude (Figures 4, 7A,B). The velocity modulation can be evaluated independently of saccadic amplitude and without sacrificing the amplitude sensitivity. For example, the velocity change under a given task condition (e.g., rewarded saccade) is readily distinguished from that under a different task condition (e.g., unrewarded saccade), as the comparison of the velocity modulation was conducted at the same amplitude bin of the same direction. In addition, the methodology conserved the data sampling of saccadic velocities (i.e., reduction of N). This feature is useful for human studies, especially when the test duration is limited.

There are alternative quantification methods that circumvent the problems of amplitude-velocity coupling. One approach is the statistical application of the analysis of covariance, which assesses the change of saccadic velocity while isolating the impact of saccadic amplitude (Bahill et al., 1975; Chen et al., 2013). This method is useful concerning the rate (slope) and magnitude (intercept) of the velocity modulation. Note that both measures can be readily computed based the mathematically equivalent equations described in this study (see Equations 1, 2, Materials and Methods). A different approach is based on the task design of fixed visual target displacements (e.g., Robinson, 1964; Collewijn et al., 1988; Takikawa et al., 2002; Xu-Wilson et al., 2009). In essence, this approach can be considered comparable to the methodology of this study, except that the bin width of saccadic amplitude is significantly large. It is known that the primary saccadic amplitude often varies significantly even if the target displacement is fixed (Becker and Fuchs, 1969). It is also known that the saccades bound for different but adjacent targets may have identical amplitudes, whereas the saccades bound for the same target may have different amplitudes (He and Kowler, 1989). Hence, it is difficult to evaluate saccadic velocities without considering the variability of saccadic amplitudes. To apply this method, it is desirable to have substantial velocity samples with robust modulation (e.g., Robinson, 1964; Collewijn et al., 1988; Takikawa et al., 2002; Xu-Wilson et al., 2009).

We addressed only the issues concerning saccadic velocities. Saccadic (amplitude) gain was out of scope of the present study. To examine the modulation of saccadic gain, one has to properly control target displacement, salience, and perhaps balancing the trade-off between speed and accuracy. It is conceivable that reward expectation modulates saccadic gain, as suggested by past studies (Shadmehr, 2010; Louie et al., 2011; Madelain et al., 2011).

### Nasal-temporal velocity asymmetry as saccadic habit

Saccadic velocities vary depending on saccadic directions; abducting (temporal) saccades tend to be faster than adducting (nasal) saccades (Robinson, 1964; Collewijn et al., 1988; Figures 3, 7). This nasal-temporal velocity asymmetry is thought to be a unique, built-in characteristic, as it is present regardless of whether the saccades are reflexive (Robinson, 1964) or voluntary (Collewijn et al., 1988; this study). This implies that the control is likely intrinsic to the saccade generator or is regulated by the structures utilized by both reflexive and voluntary saccades, for instance, at the level of or downstream from the superior colliculus. This implication is consistent with our finding that the velocity asymmetry was highly stable across the test order, i.e., resistant to the sensorimotor modulations that occurred across series of saccades (Figure 7B). This was in agreement with the other finding that the velocity asymmetry is resistant to reward modulation (Figures 8C,D).

Similar to the left-right handiness in skeletal movements, the nasal-temporal velocity asymmetry of saccades could be biased temporally (Robinson, 1964; Collewijn et al., 1988; this study, the majoirty (5/7) of subjects), nasally (Figure 7B, subject M7) or neither (Figure 7B, subject F3). A recent fMRI study found that the blood-oxygen-level dependent signal in response to 8-Hz checkerboards was stronger for temporal than nasal visual stimuli (Sylvester et al., 2007). This nasal-temporal evoked visual response was present in the superior colliculus, not in the lateral geniculate nucleus or the visual cortex. This suggests that the observed nasal-temporal asymmetry may be coupled with visual orienting (Rafal et al., 1991), as opposed to solely visual processing (cf. Fahle and Schmid, 1988). It is interesting to note that the nasal-temporal velocity asymmetry was stronger under monocular than binocular viewing conditions (Johannesson et al., 2012).

Our finding that the reward modulation of saccadic velocities was independent of the magnitude of this nasal-temporal velocity asymmetry (Figures 8C,D) deserves to be elucidated in future studies. We suggest a parsimonious explanation: the cognitive (reward) regulation of saccadic velocities is independent from the intrinsic regulation of saccadic velocities.

### Conflict of interest statement

The authors declare that the research was conducted in the absence of any commercial or financial relationships that could be construed as a potential conflict of interest.
